# Population pharmacokinetics and target attainment of ciprofloxacin in critically ill patients

**DOI:** 10.1007/s00228-020-02873-5

**Published:** 2020-04-19

**Authors:** Alan Abdulla, Omar Rogouti, Nicole G. M. Hunfeld, Henrik Endeman, Annemieke Dijkstra, Teun van Gelder, Anouk E. Muller, Brenda C. M. de Winter, Birgit C. P. Koch

**Affiliations:** 1 Department of Hospital Pharmacy, Erasmus University Medical Center, P.O. Box 2040, 3000 CA Rotterdam, The Netherlands; 2 Department of Intensive Care, Erasmus University Medical Center, Rotterdam, The Netherlands; 3 Department of Intensive Care, Maasstad Hospital, Rotterdam, The Netherlands; 4 Department of Clinical Pharmacy & Toxicology, Leiden University Medical Center, Leiden, The Netherlands; 5 Department of Medical Microbiology and Infectious Diseases, Erasmus University Medical Center, Rotterdam, The Netherlands; 6 Department of Medical Microbiology, Haaglanden Medical Center, The Hague, The Netherlands

**Keywords:** Population pharmacokinetics, Ciprofloxacin, Critically ill patients, Target attainment, NONMEM

## Abstract

**Purpose:**

To develop and validate a population pharmacokinetic model of ciprofloxacin intravenously in critically ill patients, and determine target attainment to provide guidance for more effective regimens.

**Methods:**

Non-linear mixed-effects modelling was used for the model development and covariate analysis. Target attainment of an ƒAUC_0–24_/MIC ≥ 100 for different MICs was calculated for standard dosing regimens. Monte Carlo simulations were performed to define the probability of target attainment (PTA) of several dosing regimens.

**Results:**

A total of 204 blood samples were collected from 42 ICU patients treated with ciprofloxacin 400–1200 mg/day, with median values for age of 66 years, APACHE II score of 22, BMI of 26 kg/m^2^, and eGFR of 58.5 mL/min/1.73 m^2^. The median ƒAUC_0–24_ and ƒC_max_ were 29.9 mg•h/L and 3.1 mg/L, respectively. Ciprofloxacin pharmacokinetics were best described by a two-compartment model. We did not find any significant covariate to add to the structural model. The proportion of patients achieving the target ƒAUC_0–24_/MIC ≥ 100 were 61.9% and 16.7% with MICs of 0.25 and 0.5 mg/L, respectively. Results of the PTA simulations suggest that a dose of ≥ 1200 mg/day is needed to achieve sufficient ƒAUC_0–24_/MIC ratios.

**Conclusions:**

The model described the pharmacokinetics of ciprofloxacin in ICU patients adequately. No significant covariates were found and high inter-individual variability of ciprofloxacin pharmacokinetics in ICU patients was observed. The poor target attainment supports the use of higher doses such as 1200 mg/day in critically ill patients, while the variability of inter-individual pharmacokinetics parameters emphasizes the need for therapeutic drug monitoring to ensure optimal exposure.

**Electronic supplementary material:**

The online version of this article (10.1007/s00228-020-02873-5) contains supplementary material, which is available to authorized users.

## Introduction

Patients admitted to the intensive care unit (ICU) often need antibiotic therapy to treat infections. Timely and adequate antimicrobial treatment is essential for good clinical outcome, preventing the spread of antibiotic resistance and containing the economic impact of infections [1–4]. ICU patients represent a highly heterogeneous population with significant differences in the distribution of patients’ ages, severities of illness, durations of admission, and outcomes [5]. Due to the large variability in these patients, a “one-dose-fits-all” approach seems undesirable. Furthermore, dosing of many antibiotics was designed in an era with more susceptible micro-organisms and in healthy volunteers or patients with mild to moderate severities of illness, with reasonably predictable pharmacokinetic (PK) parameters. The pathophysiological changes in critically ill patients can cause substantial PK changes, such as an increased volume of distribution, decreased protein binding, and changes in elimination rate [6–8]. PK changes in critically ill patients often result in insufficient exposure, which may contribute to inadequate bacterial eradication, an increased chance of antibiotic resistance, and excess morbidity and mortality rates [8–11].

Ciprofloxacin, a fluoroquinolone antibiotic, has a wide spectrum of antimicrobial activity and is frequently used for various infections as monotherapy or in combination with other antibiotics [12]. The bactericidal action of ciprofloxacin is characterized by a rapid concentration-dependent activity against many gram-negative aerobic bacteria and to a lesser extent against gram-positive bacteria [13]. Ciprofloxacin is eliminated by various mechanisms (renal, hepatic, and transintestinal) [14].

The ratio of the area under the drug serum concentration–time curve over 24 h at steady state and the minimal inhibitory concentration (AUC_0–24_/MIC) is a good predictor for ciprofloxacin efficacy. The pharmacodynamic target (PDT) for optimal outcome for ciprofloxacin is AUC_0–24_/MIC ≥ 125, or ≥ 100 for the unbound (free) drug concentration (ƒAUC_0–24_/MIC) [11, 15–17]. The probabilities of microbiological and clinical cure for ciprofloxacin AUC_0–24_/MIC < 125 are poor (26% and 42%, respectively), compared with AUC_0–24_/MIC ≥ 125 where the probabilities are 80% (*p* < 0.005) and 82% (*p* < 0.001), respectively [15]. In addition, C_max_/MIC ratio 8–10 is suggested to be particularly important to prevent the emergence of resistance [18]. However, it is a challenging task to formulate general dose adjustments for critically ill patients and a validated approach for dose adjustment is not currently available. Therapeutic drug monitoring (TDM) combined with a population pharmacokinetic (popPK) model can be used to interpret the complex PK in critically ill patients and support in optimizing individual dosing to improve attainment of the predefined targets. To date, different ciprofloxacin popPK models have been developed for ICU patients [14, 19–23]. In most models, the study populations were exposed to daily doses of ≤ 1200 mg/day, and in only three models Monte Carlo simulations were performed to define dosing regimens that increase the probability of target attainment (PTA) [14, 23, 24]. The current study is one of the largest multi-centre trials describing detailed ciprofloxacin population pharmacokinetics in ICU patients. In contrast to the majority of the previous studies, pharmacokinetic data was obtained based on data from a broad dosage range (400–1200 mg).

The objective of this study was to develop a popPK model to determine inter-individual PK variability, the influence of patient characteristics, and the PTA of different high dosing regimens using Monte Carlo simulations in ICU patients. Furthermore, the model in the current study is described in detail and comprehensively validated. Such knowledge is essential for implementing model-based dosing to optimize ciprofloxacin exposure in critically ill patients.

## Methods

### Study design and population

The popPK model for ciprofloxacin was developed based on data from a two-centre, prospective, observational PK/PD study in the ICU departments of the Erasmus Medical Centre and Maasstad Hospital, Rotterdam, the Netherlands (EXPAT study). All patients admitted to the ICU between January and December 2016 and treated with ciprofloxacin were assessed for inclusion. Eligible for enrolment were patients aged ≥ 18 years, receiving intravenous ciprofloxacin, and treatment aimed for at least 3 days. Exclusion criteria were antibiotic cessation before sampling and burn wound patients admitted to the ICU. The initiation of ciprofloxacin, dosage, and duration of therapy were at the discretion of the attending physician.

### Blood sampling and assays

On day two after start of ciprofloxacin administration, blood samples were collected before administration (trough concentration), 15–30 min after the end of the infusion (peak concentration), 1 and 3 h after infusion, and just before the start of the next dose (second trough concentration). The exact sampling time and the dosage administered were recorded. Blood samples were stored at 2–8 °C to maintain integrity, and centrifuged at 3000 rpm for 6 min within 24 h of collection. The plasma was transferred to cryo-vials for frozen storage (− 80 °C) until analysis. Plasma concentrations were determined by a multi-analyte UPLC-MS/MS. The calibration curves were linear from 0.04 up to 5.0 mg/L, giving a correlation coefficient *r*^2^ = 0.999. Samples with a concentration above 5.0 mg/L were diluted according to a standard dilution protocol. The method was comprehensively validated according to the Food and Drug Administration (FDA) guidance on bioanalytical method validation [25]. Observed concentrations were corrected for protein binding (ƒAUC = AUC ∙ 0.7), using an average plasma protein binding (PPB) value of 30% in critically ill patients [26, 27].

### Model building

The popPK model was built by using non-linear mixed-effect modelling (NONMEM®, version 7.2, ICON Development Solutions, Ellicott City, MD, USA). The graphical user interface (GUI) Pirana [28] (version 2.7.0) was used for model management, execution, output generation, and interpretation of results. Pirana was also used as the GUI for PSN (version 4.7.0) and Xpose (version 4.5.3), and R-studio was used in combination with Pirana for graphical visualization. The data were analyzed using the first-order conditional estimation method with interaction (FOCE-I).

### Structural model

For the initial popPK model, one-, two-, and three-compartment models were tested to fit the ciprofloxacin plasma concentration data and calculate the clearance (CL), volume of distribution of the central and peripheral compartment (respectively *V*_c_ and *V*_p_), and the transfer of ciprofloxacin between the central and peripheral compartment (*Q*). The model quality and the selection were based on the precision with which the model parameters were estimated, objective function value (OFV), shrinkage values, and visual inspection of the goodness-of-fit-plots. The inter-individual variability (IIV) was estimated on each parameter by using an exponential model. For these parameters, the shrinkage was also calculated to identifying and quantifying whether an overfit is taking place. A shrinkage below 20% was considered acceptable [29]. The residual variability was incorporated as a combined proportional and additive model. The IIV of the parameters, for example clearance, can be described by the following equation:

1 $$ {\mathrm{CL}}_j={\mathrm{CL}}_{\mathrm{pop}}\times \exp \left({\eta}_{\mathrm{CL}}\right) $$

CL_*j*_ is the clearance of the _*j*_th individual and it is described by the clearance of the mean population (CL_pop_) and variability of mean clearance and the clearance of the _j_th individual (*η*_CL_). *η*_CL_ is normally distributed with an average of zero and a variance of *ω*^2^, shortly noted as *η* = *N*(0,*ω*^2^). To further refine the model, the omega block option was used for assessment of covariance between random effects.

### Covariate analysis

After the selection of the structural model, covariates were added to the model. These covariates were selected based on the possibility that they could explain the IIV in parameter estimates. This was based on relevant physiological and clinical explanations or evidence from previous research [14, 19–21, 30]. Covariates that were tested were serum creatinine, estimated glomerular filtration rate (eGFR), serum albumin, body mass index (BMI), weight, sex, renal replacement therapy (RRT), and age. The continuous covariates were normalized to the population median and the categorical covariates were transformed to binary covariates, respectively Eqs. 2 and 3.

2 $$ {\theta}_i={\theta}_{\mathrm{pop}}\times {\left(\frac{{\operatorname{cov}}_i}{{\operatorname{cov}}_m}\right)}^{\theta_{cov}} $$

3 $$ {\theta}_i={\theta}_{\mathrm{pop}}\times {\theta_{\mathrm{cov}}}^{{\operatorname{cov}}_i} $$

*θ*_*i*_ represents the individual predicted value of any parameter calculated by the model with a covariate value cov_*i*_. *θ*_pop_ represents the population estimate for *θ*_*i*_, cov_*m*_ is the median covariate value, and *θ*_cov_ is the covariate effect. For Eq. 3, the covariate value is either 1 or 0. The covariates were individually added to the model and then deleted one by one according to the forward inclusion-backward elimination method [31]. For the initial covariate step, a decrease in OFV of at least 3.84 (*p* < 0.05 with 1 degree of freedom) from the structural model was required for the covariate to be included. Subsequently, all significant covariates were included simultaneously to the structural model and were deleted one by one. A stricter statistical significance of *p* < 0.001 was applied in the backward elimination step (OFV > 10.83).

### Model evaluation

The evaluation of the model was done using statistical and graphical tools, including goodness-of-fit plots. Furthermore, the robustness of parameter estimates from the final model was tested using a bootstrap analysis. For the bootstrap, the dataset was resampled 1000 times to asses if the model was appropriate. Visual predictive checks (VPCs) were executed to evaluate the model [32]. A normalized prediction distribution error (NPDE) analysis, which is a simulation-based diagnostic tool that can be used to evaluate models which have different dosage regimens, was also used to evaluate the final model [33].

### Pharmacodynamic target

To calculate the ƒAUC_0–24_/MIC and ƒC_max_/MIC ratios, the clinical breakpoint of 0.5 mg/L from the EUCAST database was used [34]. This is the highest MIC from which it can be expected that ciprofloxacin under standard conditions is effective for *Enterobacteriaceae*, *Pseudomonas* spp., *Acinetobacter* spp., *Haemophilus influenzae*, *Moraxella catarrhalis*, and *Neisseria* spp. [34]. To assess the suitability of the empirical fixed dosing regimens considering ƒAUC_0–24_/MIC ≥ 100 and ƒC_max_/MIC ≥ 8, a MIC distribution of 0.0312–8 mg/L was tested. For the targeted treatment at different MICs, the wild-type population distribution of *Pseudomonas aeruginosa* and the epidemiological cut-off (ECOFF) value from the EUCAST database were used [34].

### Dosing simulations

To generate data for target attainment analyses, Monte Carlo simulations (*n* = 5000) were performed to define the PTA for ciprofloxacin 400 mg twice daily (q12h), three times daily (q8h), four times daily (q6h), 600 mg q12h, and q8h for the non-protein bound fraction using MicLab236b (Medimatics, Maastricht, the Netherlands). Monte Carlo simulations use a simulation platform to expand the sample size of a study to provide predictions of the likely result of different therapeutic approaches, such as altered drug dose or frequency, on the achievement of therapeutic targets [35].

## Results

### Study population

A total of 42 patients were included. Among these patients, the prescribed daily dose was 400 mg q24h in 3 patients, 400 mg q12h in 25 patients, and 400 mg q8h in 14 patients, administered as an infusion over 30–60 min. In total, 204 plasma concentrations were available, an average of 4.9 samples per patient. Other baseline characteristics of the study population were median eGFR 58.5 mL/min/1.73 m^2^, albumin 25 g/L, C-reactive protein 139.5 mg/L, Acute Physiology and Chronic Health Evaluation (APACHE) II score 22, Sequential Organ Failure Assessment (SOFA) score 13, and a mortality rate at day 30 of 23.8%. A summary of baseline patient characteristics is presented in Table 1.

**Table 1:** Summary of baseline patient demographic and clinical characteristics

Characteristics	*n* = 42
Demographic data	
Sex (male/female)	25/17
Age (years)	65.5 (56–71)
Body weight (kg)	80 (64–90)
Height (cm)	173 (165–181)
BMI (kg/m^2^)	26 (17.8–46.3)
Primary diagnosis	
Respiratory	20 (47.6)
Cardiovascular	5 (11.9)
Gastrointestinal	5 (11.9)
Sepsis	8 (19.0)
Neurological	2 (4.8)
Other	2 (4.8)
Clinical data	
APACHE II	22 (20–26)
SOFA score	13 (9–16)
Length of ICU stay (days)	8.5 (4.0–28.3)
30-day survival	32 (76.2)
Biological data	
Serum creatinine (μmol/L)	90 (70–153)
Serum creatinine (mg/dL)	1.0 (0.8–1.7)
eGFR (mL/min/1.73m^2^)	58.5 (32–101)
Albumin (g/L)	25 (22–29)
C-reactive protein (mg/L)	139.5 (71–194)
Leukocytes (×10^9^/L)	13.4 (9.7–20.8)
Extra-corporal circuits	
CVVH	10 (23.8)
Pharmacological data	
ƒAUC_0–24_ (mg·h/L)	29.9 (19.6–42.1)
ƒCmax (mg/L)	3.1 (2.4–4.0)
Concomitant antibiotics	
Cefotaxime	27 (64.3)
Metronidazole	13 (31.0)
Gentamicin	6 (14.3)
Amoxicillin	3 (7.1)
Doxycycline	1 (2.4)
Cefuroxime	1 (2.4)
Ceftazidime	1 (2.4)
Co-trimoxazole	1 (2.4)

Data are expressed as *n* (%) or median (IQR)

*M*, Male; *F*, Female; *BMI*, Body Mass Index; *SOFA score*, Sequential Organ Failure Assessment score; *APACHE II*, Acute Physiology and Chronic Health Evaluation II; *ICU*, Intensive Care Unit; *eGFR*, estimated Glomerular Filtration Rate, calculated using the Modification of Diet in Renal Disease (MDRD) formula; *CVVH*, Continuous Venovenous Hemofiltration

### Pharmacokinetic parameters

**Fig. 1:**
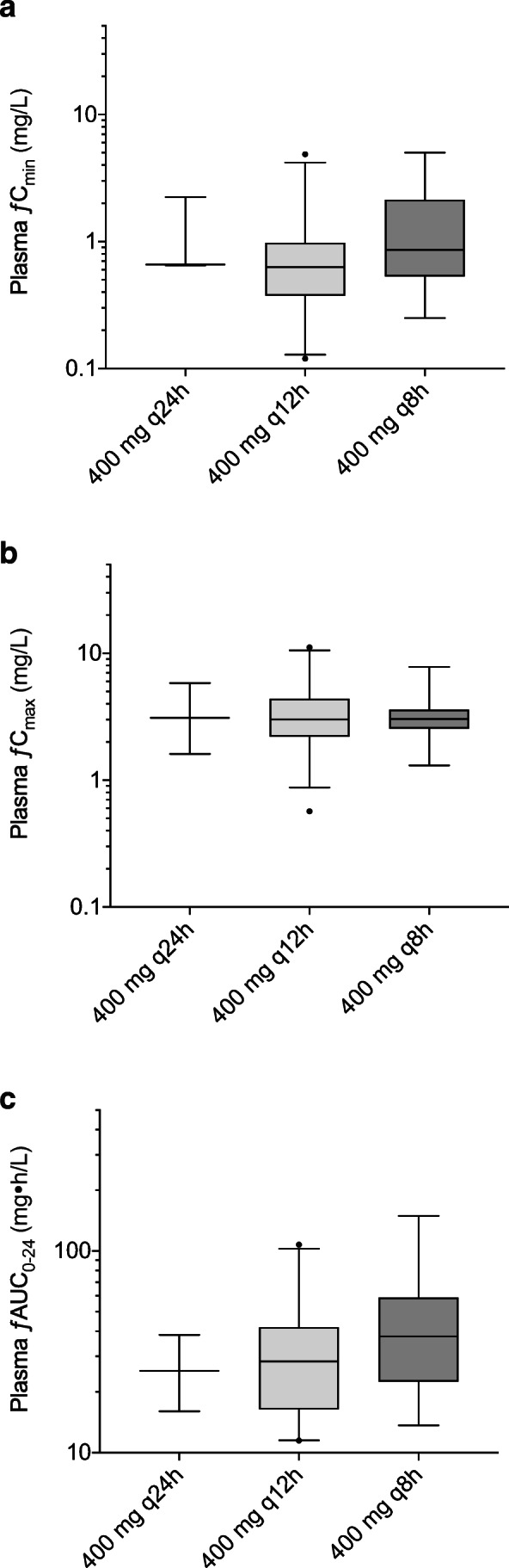
Box (median, 25th and 75th percentiles) and whisker (10th and 90th percentiles) plots of free (**a**) trough (ƒC_min_), (**b**) peak (ƒC_max_) plasma concentrations, and (**c**) area under the plasma concentration versus time curves (ƒAUC_0–24_) of ciprofloxacin observed in severely ill patients treated with 400 mg one (q24h), two (q12h), and three (q8h) times daily. Filled circles are outliers

Box and whiskers plots of plasma ƒC_min_, ƒC_max_, and ƒAUC_0–24_ for the different dosing groups are shown in Fig. 1. Plots of the total trough and peak plasma concentrations are presented in the Supplemental material (Fig. S1). In the 400 mg q24h, q12h, and q8h groups, the mean ƒC_max_ were 3.10, 3.02, and 3.05 mg/L, respectively, and the mean ƒAUC_0–24_ were 26.6, 34.2, and 46.8 mg•h/L, respectively.

### Final model

The data was best described by a two-compartment model and the residual error was described by a combined additive and proportional error model. IIV was included on CL, *V*_c_, and *Q* and significantly improved the model (*p* < 0.05). The omega block construction between CL and *V*_c_ was found to improve the model and was maintained during the model building process. The parameter estimations of the final model are presented in Table 2. The mean serum elimination half-life was 6.96 h.

**Table 2:** Parameter estimates of the final model and bootstrap analysis

Parameter	Final model	Bootstrap of the final model	
		Median	95% CI
CL (L/h)	25.4 (11)	25.8	20.6–30.6
*V*_c_ (L)	91.1 (13)	88.8	61.8–110.7
*V*_p_ (L)	164 (15)	159.8	120.1–216.2
*Q* (L/h)	91.9 (10)	94.3	77.2–128.3
IIV (%)			
CL	67.8 (12) [1]	66.0	50.7–81.4
Vc	51.0 (22) [13]	37.7	11.1–55.3
Residual variability (%)			
Proportional	15.3 (48) [18]	15.4	0.1–24.7
Additional	14.3 (55) [18]	14.0	0.1–27.7

The relative standard error (expressed as percentages) is given in round brackets, and the shrinkage (expressed as percentages) is given in square brackets

*CL*, Clearance of ciprofloxacin; *V*_*c*_, Volume of distribution in the central compartment; *V*_*p*_, Volume of distribution in the peripheral compartment; *Q*, inter-compartmental clearance; *IIV*, Inter-Individual Variability

### Covariate analysis

The base two-compartment model with IIV on CL, *V*_c_, and *Q* was used as a reference model for the covariate analysis. After graphically selecting covariates for analysis, a forward selection of covariates followed by a backward elimination was carried out. None of the covariates were found to correlate significantly.

### Model evaluation

**Fig. 2:**
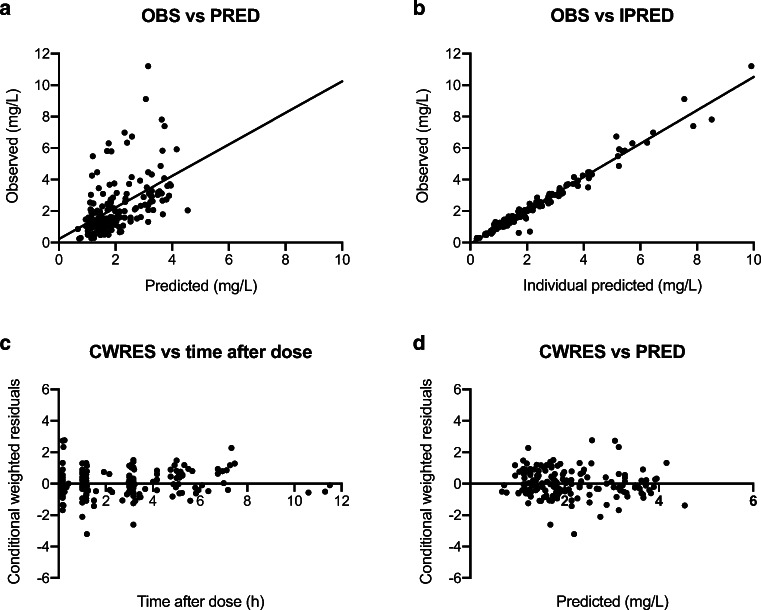
Goodness-of-fit plots of the final model. (**a**) Observed concentration (OBS) plotted against predicted concentration (PRED). (**b**) OBS plotted against the individual predicted concentration (IPRED). (**c**) Conditional weighted residuals plotted against time after dose. (**d**) CWRES plotted against PRED. The line in A and B represents the line of identity

**Fig. 3:**
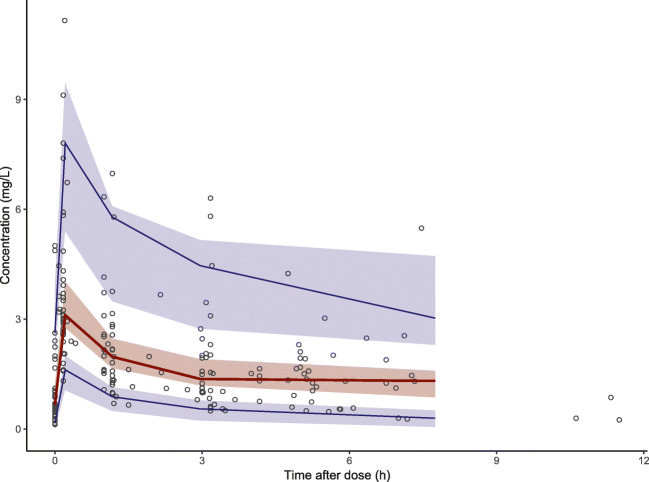
Observed ciprofloxacin concentration–time data and the visual predictive check (VPC) of the final model. The blue brackets are the observed concentrations. The red line is the observed median and the two blue lines are the 5th and 95th percentiles of the observed data. The red shaded area is the 95% CI of the model-predicted median and the blue shaded areas are the 95% CIs of the model-predicted 5th and 95th percentiles

The population predictions and individual predictions of the final model were evenly distributed around the line of unity when plotted against the observations, as shown in Fig. 2. The conditional weighted residuals were normally distributed over the *x*-axis when plotted against the time after dose and concentration (Fig. 2). To assess the uncertainty of parameters, a bootstrap analysis with 1000 runs was performed to calculate the 95% percentile range of the final PK parameters. The median values and 95% CIs of the performed bootstrap analysis are shown in Table 2. The VPC of the final model showed good model predictability. The median observations, represented by the red line in the middle, were lying within the 95% CI of the model predictions, represented by the red shaded areas, thereby demonstrating adequate fit of the model (Fig. 3). The NPDE analysis are illustrated in the Supplement material, both graphs in Fig. S2 did not deviate significantly from a normal distribution and with the majority of the NPDEs lying between the values − 2 and 2, the model was considered appropriate.

### Pharmacodynamic target attainment

The percentage of patients achieving the PDT ƒAUC_0–24_/MIC ≥ 100 and ƒC_max_/MIC ≥ 8 in the three different ciprofloxacin intravenous dosing regimens groups were calculated for different MIC values (Supplemental Fig. S3). Of all patients, for the breakpoints 0.25 and 0.5 mg/L, the ƒAUC_0–24_/MIC ≥ 100 target was achieved in 61.9% and 16.7% of the patients, respectively. Although there was a difference in achieving the PDT between 400 mg q12h (12.0%) and q8h (28.6%) groups for the breakpoint 0.5 mg/L, this numerical difference did not reach statistical significance (*p* = 0.19). This can be explained by the large variation and overlap of the ƒAUC_0–24_ in both dosing groups (Fig. 1C). In addition, there was significant difference in baseline eGFR between the three dose groups. The median eGFR was 28.0, 52.0, and 82.5 mL/min/1.73 m^2^ for the 400 mg q24h, q12h, and q8h groups, respectively. Furthermore, the ƒC_max_/MIC concentration ratios of ≥ 8 for the breakpoint of 0.25 and 0.5 mg/L were realized in 34 (81.0%) and 11 (26.2%) of patients, respectively.

### Dosing simulations

**Fig. 4:**
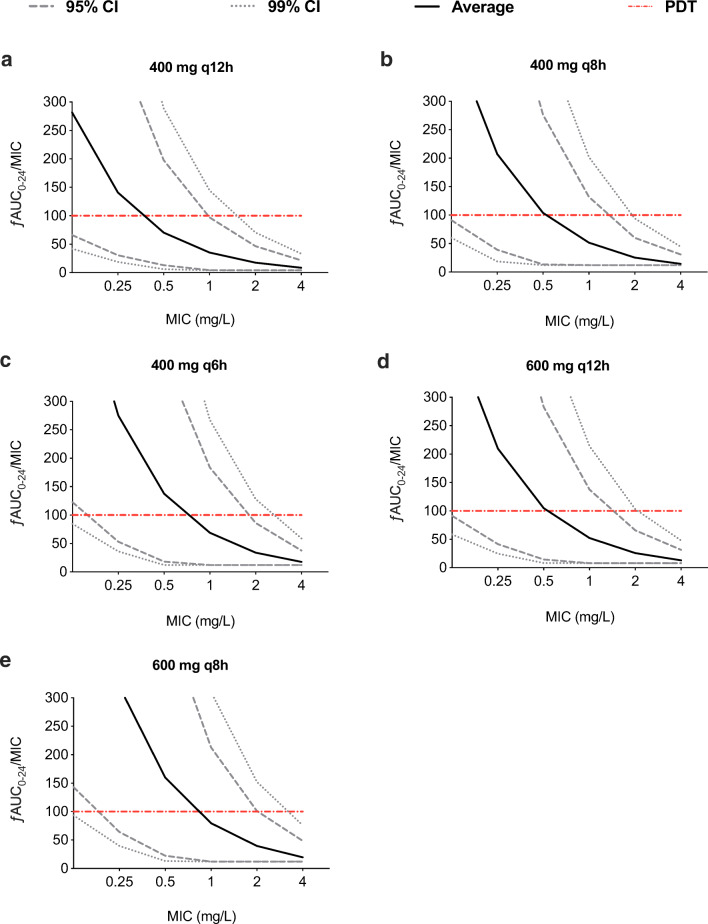
Probabilities of target attainment (PTA) for ciprofloxacin 400 mg (**a**) q12h, (**b**) q8h, (**c**) q6h, 600 mg (**d**) q12h and (**e**) q8h. Dotted lines indicate the 95% and 99% confidence intervals (CI). The red reference line represents the pharmacodynamic target of ƒAUC_0–24_/MIC ≥ 100

The popPK parameters from the final model were used to conduct Monte Carlo simulations to assess the target attainment. Figure 4 shows the ƒAUC_0–24_/MIC ≥ 100 as a function of the MIC for several dosing regimens. In order to achieve optimal exposure (ƒAUC_0–24_/MIC ≥ 100) at an MIC of 0.5 mg/L, a dose of 1200 mg/day was required on average (Fig. 4C–E). Since the 95% and 99% CIs are wide, even in the highest dosage regimes, a substantial proportion of the population does not achieve the PDT.

## Discussion

In this study, we present the results of intravenous ciprofloxacin PK modelling in 42 critically ill patients. Our popPK model of ciprofloxacin was best described by a two-compartment model, similar to previous studies [14, 19, 21, 23]. The final model was comprehensively evaluated by using NPDE analysis and VPCs. Our ICU population is very heterogeneous, with a great variety of primary diagnosis, and clinical and biological characteristics (Table 1). This variability is represented in the model by the relatively high IIV. The high IIV in *V*_c_ cannot be explained by fluid shifts, since ciprofloxacin volume of distribution in critically ill patients does not change over time [36]. Van Zanten et al. [21] also found up to fivefold differences in volumes of distribution. However, they concluded that patient biometry or excessive volume loading could not explain the differences in volumes of distribution.

Various covariates that could provide information on PK of ciprofloxacin in critically ill patients were tested. However, at the end of the model-building process, no significant covariates were found on *V*_c_, CL, and *Q*. The lack of significant covariates can partly be explained by the high variability in the PK of ciprofloxacin in ICU patients as recurrently described in the literature [15, 19–21]. The estimated renal function in our population has a wide distribution, due to the presence of patients with and without acute or chronic kidney injury, and RRT (Table 1). Nevertheless, both impaired renal function and the presence of CVVH did not have a significant influence on the model and were not incorporated in the final model as a covariates. This implies that plasma ciprofloxacin exposure cannot be predicted by using serum creatinine in our ICU population. Using the creatinine to estimate renal function is known to poorly predict actual renal function, as it is affected by factors other than renal function [37]. However, in other popPK studies, the creatinine clearance was found to be a significant relevant covariate [19, 20]. Variability in ICU population characteristics (e.g. admission diagnosis and disease state) can be a likely explanation for this difference in influence of serum creatinine. While in our study, we included septic and non-septic patients (Table 1). Conil et al. [19] included only septic patients and found a significant influence of CL_Cr_ on *k*_el_. Forrest et al. [20] found a significant relationship between total ciprofloxacin clearance and CL_Cr_ estimated by the Jelliffe formula, mostly in patients with lower respiratory tract infection. Additionally, when ciprofloxacin renal clearance is compromised, the transintestinal elimination route is frequently described in the literature in humans and animals as the main compensatory elimination route [38–40]. In septic patients, Jones et al. [39] showed that only those patients who had liver or bowel pathology in addition to renal failure had a significantly higher serum concentration than all other patients. Nevertheless, dose reduction or interval extension have been proposed in the literature for patients with only impaired renal function [41–43]. Concurrently, the results of various studies show the importance of adequate dosing in ICU patients, suggesting not reducing the dose of ciprofloxacin in patients with impaired renal function [21, 39, 44]. In addition, no significant renal accumulation of ciprofloxacin in patients with an impaired renal function was observed [44, 45]. This supports ciprofloxacin TDM when dose reduction is considered in patients with impaired renal function to avoid underdosing.

Our study shows that the PDTs are seldomly reached using ciprofloxacin standard (800 mg/day) and high exposure (1200 mg/day) dosing in ICU patients. The PDT was only achieved in 16.7% of all patients at the clinical breakpoint of 0.5 mg/L. These findings are consistent with results from previous studies on exposure of ciprofloxacin in critically ill patients [15, 21, 22]. However, a breakpoint of 0.5 mg/L is only applicable when high exposure dosing is used (≥ 1200 mg/day), covering *P. aeruginosa* infection [34]. For microorganisms categorized as susceptible for standard dosing regimen, MICs ≤ 0.25 mg/L are appropriate to assess the probability of therapeutic success.

In this study, the PTA was simulated to identify ciprofloxacin intravenous dosing regimens that might better enable optimal target attainment. The simulations indicate that for this population of ICU patients, the variation is significant, and a PTA of at least 95% is only obtained for MIC values ≤ 0.25 mg/L. To reach a target of anƒAUC_0–24_/MIC ≥ 100 for these MICs, at least 1200 mg/day is required to ensure optimal exposure (> 95% PTA) in ICU patients (Fig. 4). For directed therapy against *P. aeruginosa* (an MIC of 0.5 mg/L) in patients with septic shock, even a higher dose of 600 mg q8h has been recommended to achieve adequate target attainment [23]. Furthermore, Roberts et al. [23] simulated an 800 mg loading dose, followed by 400 mg q8h doses, and demonstrated an increase in PTA on day 1 of therapy by 35–45%, compared with standard 400 mg q8h.

However, in clinical practice, the regular ciprofloxacin dosing falls within a relatively narrow range of 800–1200 mg/day, is not adjusted for body weight, and only moderately increased for severe infections in the ICU. Higher doses may also increase the risk of potential adverse events. Given that there is high degree of variety in PK of ciprofloxacin in critically ill patients, as demonstrated here, it follows that TDM in ICU patient is strongly recommended to increase the likelihood of therapeutic target achievement and avoid unnecessary high concentrations.

In this study, five samples per interval were used to estimate the AUC_0–24_, but in clinical practice this is not convenient. Thus, for TDM in clinical practice, we recommend a peak sample, or every second sample next to a trough sample to estimate the C_max_ and AUC_0–24_ with sufficient accuracy. Considering that patients with ƒAUC_0–24_/MIC ≥ 100 have the highest cure rates [15], patients with infections caused by micro-organisms at higher MICs may benefit most of TDM, as traditional dosing is likely to result in inadequate exposure in the majority of ICU patients. Inadequate antibiotic exposure in ICU patients also appears to be an important independent determinant of hospital mortality [46]. Considering the increasing resistance to ciprofloxacin worldwide, at least 1200 mg/day dosing and preferably combined with TDM is warranted in critically ill patients [21, 23, 47]. However, current dosing recommendations of > 1200 mg/day are only based on simulations and have not prospectively and externally been validated. It should also be noted that the risk of adverse events for the > 1200 mg/day dosage simulated in this study has not been investigated. Furthermore, it is not clear whether toxicity is predominantly peak or AUC driven so that potential adverse effects could be reduced by altering the number of administrations per day.

The present study shows some pitfalls that should be discussed. First, the ƒAUC was calculated assuming a PPB 30%. Measuring unbound ciprofloxacin concentrations is desirable when treating ICU patients, since the ratio of bound and unbound drugs can be subject to change because of disease characteristics in critically ill patients. However, protein binding of ciprofloxacin is too low to be clinically affected by the decrease of serum albumin for instance, making the calculation of unbound concentrations from published protein binding values acceptable. We analyzed plasma protein unbound fractions in another cohort of ICU patients [48] to clarify the clinical feasibility of calculating unbound fractions using an average PPB value. The mean fraction of ciprofloxacin unbound plasma concentrations (*n* = 36) in the range of 0.1–12.5 mg/L was 70.5% ± 4.7% SD (data not published yet). This is comparable with the calculated free fraction used in this study and previously published data [26, 27].

Second, we used ECOFF values to calculate the PTA, since the ECOFF in many situations is similar to the clinical breakpoint [49]. Due to this approach, there is a chance that PTA is underestimated in our study. However, the use of a measured MIC obtained by a single MIC determination is debatable, since routine clinical laboratories cannot determine MICs with sufficient accuracy due to the inherent assay variation in the MIC test and the variation in any MIC determination [49].

Third, we used serum creatinine concentration as a testing covariate on clearance. The creatinine clearance is the method of reference for the estimation of the GFR. However, it is not directly measured but an estimation by equation (i.e. Cockcroft-Gault or MDRD), which is not validated for critically ill patients. Changes in serum creatinine are delayed after changes in GFR, and fluid changes in critically ill patients can seriously complicate the capability of serum creatinine to detect small changes in kidney function [50, 51].

## Conclusion

Our model describes the complex PK of intravenous ciprofloxacin in critically ill patients. We found a high inter-individual variability of ciprofloxacin PK. The obtained variability of our final model parameters in combination with the presented low target attainment suggests higher initial doses of at least 1200 mg/day are needed in critically ill patients. More clinical outcome studies are necessary to support this proposal, and to support the need for therapeutic drug monitoring to ensure optimal exposure. To confirm the correlation of current PK/PD targets with optimal patient outcomes, future clinical studies should validate and evaluate outcome benefits from improved ciprofloxacin exposure using a randomized controlled trial design.

## Electronic supplementary material

ESM 1 (PDF 143 kb)
