# Supplementary material for: Population pharmacokinetics and target attainment of ciprofloxacin in critically ill patients
Source: Eur J Clin Pharmacol. 2020 Apr 19;76(7):957–67. doi: 10.1007/s00228-020-02873-5 (PMC7306030; doi:10.1007/s00228-020-02873-5)
Supplement: Supplementary file 1 — (PDF 143 kb) [file 228_2020_2873_MOESM1_ESM.pdf]

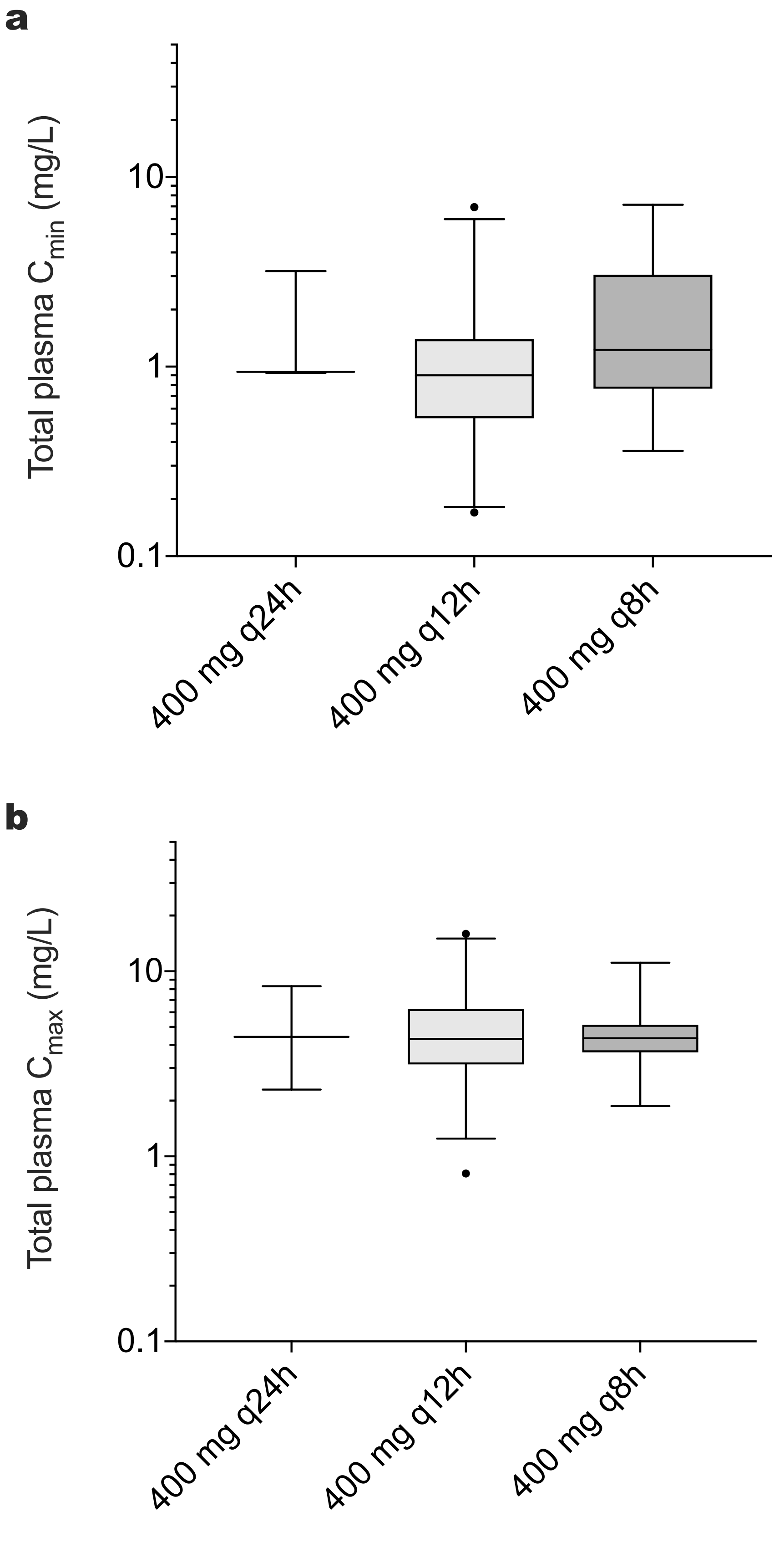

Supplemental Fig. S1. Box (median, 25th and 75th percentiles) and whisker (10th and 90th percentiles) plots of (**a**) trough (C_{min}) and (**b**) peak (C_{max}) plasma concentrations of ciprofloxacin observed in severely ill patients treated with 400 mg one (q24h), two (q12h), and three (q8h) times daily. Filled circles are outliers.

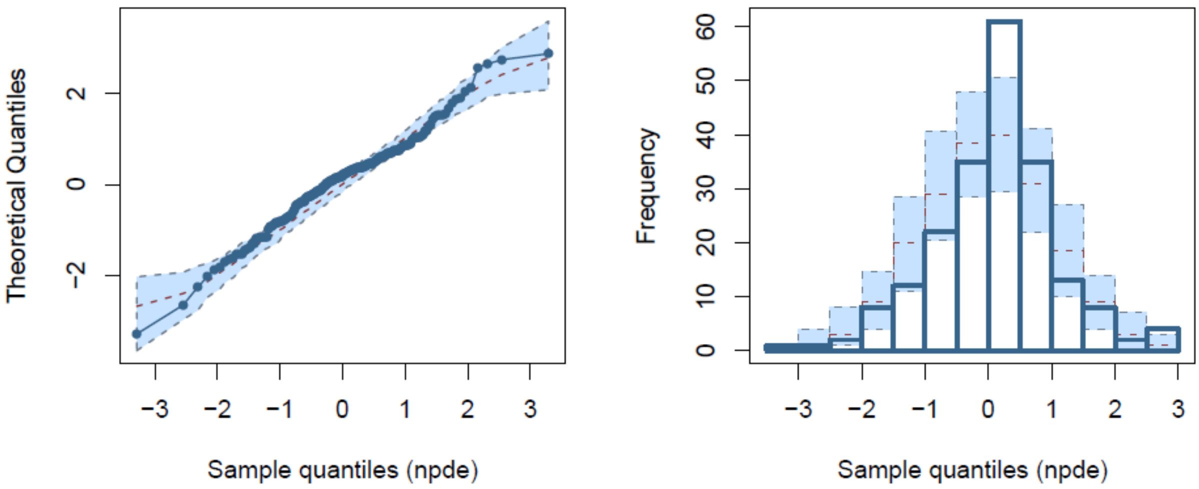

Supplemental Fig. S2. Results of the Normalized prediction distribution errors (NPDE) analysis for the ciprofloxacin final model. Quantile-quantile plot of NPDE versus expected standard normal N (0,1) distribution (left). Histogram of NPDE with the density of N (0,1) overlaid (right).

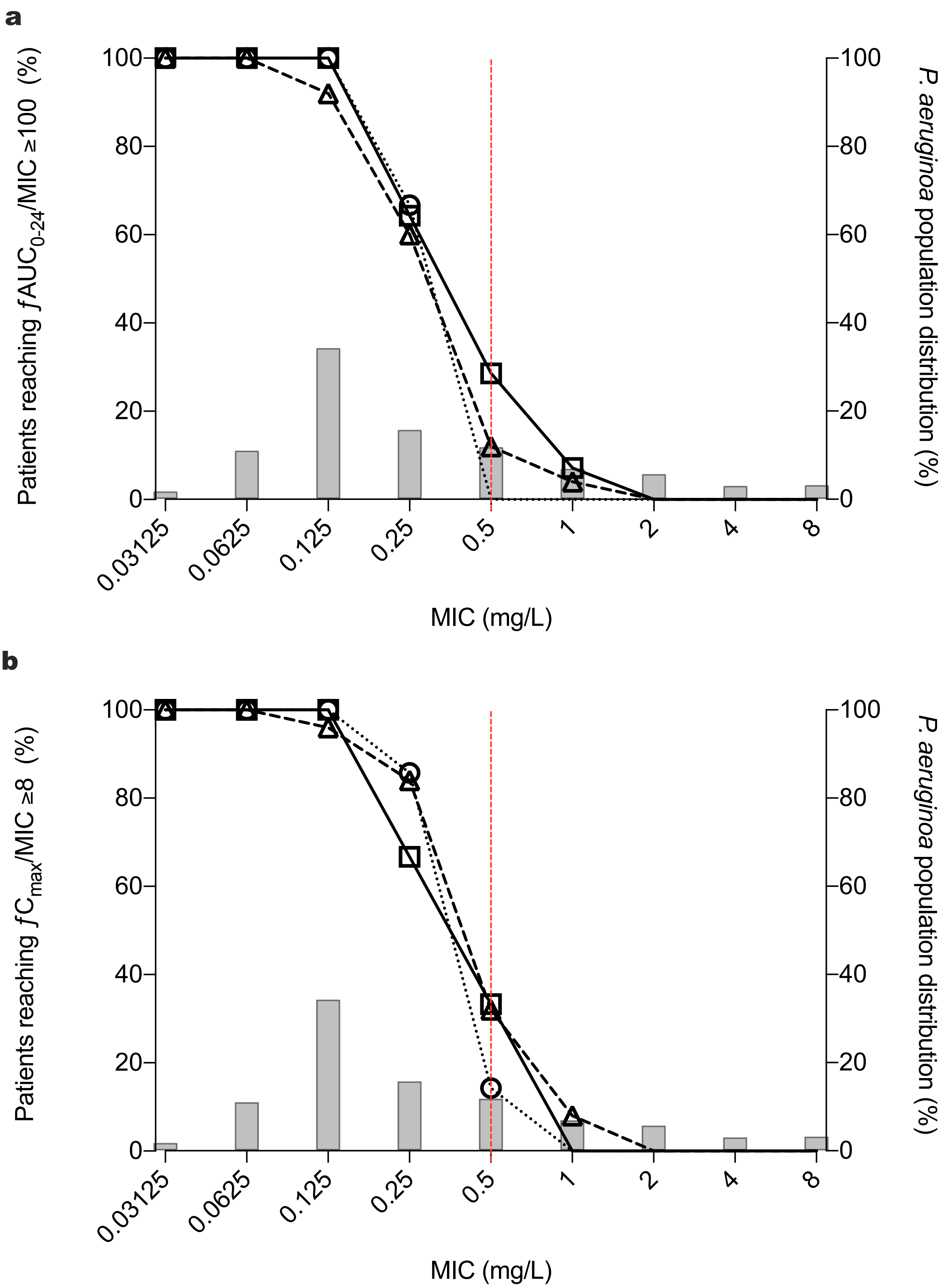

Supplemental Fig. S3. Percentage target attainment (left y-axis) for ciprofloxacin intravenous (○) 400 mg q24h, (△) 400 mg q12h, (□) and 400 mg q8h. The PDT is calculated by means of the following formula: **(a)** $fAUC_{0\text{-}24}$/MIC ≥100, where $fAUC_{0\text{-}24}$ is the estimated area under the plasma concentration–time curve during a 24 h dosing interval, **(b)** and fC_{max}/MIC ≥8. Bars represent the wild-type population distribution of *Pseudomonas aeruginosa* (right y-axis) and the red reference line is the epidemiological cut-off (ECOFF) value, as gathered from the EUCAST database.
